# Analysis of Recurrent Urinary Tract Infection Management in Outpatient Settings Reveals Opportunities for Antibiotic Stewards

**DOI:** 10.1017/ash.2021.63

**Published:** 2021-07-29

**Authors:** Marissa Valentine-King, Barbara Trautner, Roger Zoorob, George Germanos, Jason Salemi, Kalpana Gupta, Larissa Grigoryan

## Abstract

**Background:** Studies of antibiotic prescribing choice and duration have typically excluded women with recurrent UTI (rUTI), yet the Infectious Disease Society of America (IDSA) UTI treatment guidelines are applicable to recurrent and sporadic cystitis. We sought to better understand prescribing practices among uncomplicated rUTI patients in terms of choice of drug, duration of therapy, and the risk factors for receiving guideline-discordant therapy. **Methods:** We performed a retrospective database study by extracting electronic health record data from adults seen at academic primary care, internal medicine, or urology practices between November 2016 and December 2018. Inclusion criteria included having ≥2 or ≥3 *International Classification of Diseases Tenth Edition* (ICD-10) cystitis codes recorded within a 6- or 12-month period, respectively. We excluded patients with ICD-10 codes indicating any structural or functional genitourinary comorbidities, interstitial cystitis, vaginosis, compromised immune systems, or pregnancy in the prior year. Patients were also excluded if they had signs or symptoms of pyelonephritis at presentation. **Results:** Overall, 232 patients presented for 597 outpatient visits. Most were married (52.2%), non-Hispanic white (62.9%), and female (92.2%), with a median age of 58 years (IQR, 41–68). Only 21% of visits with an antibiotic prescribed for treatment consisted of a first-line therapy agent prescribed for the recommended duration. In terms of antibiotic choice, these agents were prescribed in 58.4% of scenarios, which primarily included nitrofurantoin (37.8%) and trimethoprim-sulfamethoxazole (TMP-SMX) (20.3%). Guideline-discordant choices of fluoroquinolones (28.8%), and β-lactams (11.2%) were the second and third most commonly prescribed drug categories, respectively. Multinomial logistic regression identified age (OR, 1.02; 95% CI, 1.002–1.04) or having a telephone visit (OR, 3.17; 95% CI, 1.54–6.52) as independent risk factors for receiving a β-lactam. The duration exceeded the 3-day guideline recommendation in 87.6% of fluoroquinolones and 73% of TMP-SMX (73%) prescriptions, and 61% of nitrofurantoin prescriptions exceeded the recommended 5-day duration. Multiple logistic regression analysis revealed that seeking care at a urology clinic (OR, 2.81; 95% CI, 1.59–5.17) served as an independent factor for therapy duration exceeding guideline recommendations. **Conclusions:** This retrospective study revealed shortcomings in prescribing practices in the type and duration of therapy for rUTI. rUTI as well as sporadic UTI are important targets for outpatient antibiotic stewardship interventions.

**Funding:** This investigator-initiated research study was funded by Rebiotix Inc, a Ferring Company.

**Disclosures:** None

